# A TetR family regulator of an RND efflux system that directs artemisinin resistance in *Vibrio cholerae*

**DOI:** 10.1128/msystems.00851-23

**Published:** 2023-12-19

**Authors:** In-Young Chung, Shin-Yae Choi, Hee-Won Bae, You-Hee Cho

**Affiliations:** 1 Department of Pharmacy, College of Pharmacy and Institute of Pharmaceutical Sciences, CHA University, Seongnam-si, South Korea; Georgia Institute of Technology, Atlanta, Georgia, USA

**Keywords:** artemisinin, antimalarial, antibacterial, RND efflux, VexAB, TolC, *Vibrio cholerae*

## Abstract

Artemisinin (ARS) displayed bactericidal activity against *Vibrio cholerae*. To assess the mechanistic details of its antibacterial action, we have isolated *V. cholerae* mutants with enhanced ARS resistance and identified a gene (VCA0767) whose loss-of-function resulted in the ARS resistance phenotypes. This gene (*atrR*) encodes a TetR family transcriptional regulator, and its deletion mutant displayed the reduction in ARS-induced ROS formation and DNA damage. Transcriptomic analysis revealed that the genes encoding a resistance-nodulation-cell division (RND) efflux pump operon (*vexRAB*) and the outer membrane component (*tolC*) were highly upregulated in the *artR* mutant, suggesting that AtrR might act as a negative regulator of this operon and *tolC*. Gene deletion of *vexR*, *vexB*, or *tolC* abrogated the ARS resistance of the *atrR* mutant, and more importantly, the ectopic expression of VexAB-TolC was sufficient for the ARS resistance, indicating that the increased expression of the VexAB-TolC efflux system is necessary and sufficient for the ARS resistance of the *atrR* mutant. The cytoplasmic accumulation of ARS was compromised in the *vexBtolC* mutant, suggesting that the VexAB-TolC might be the primary efflux system exporting ARS to reduce its toxicity inside of the bacterial cells. The *atrR* mutant displayed resistance to erythromycin as well in a VexR-dependent manner. This result suggests that AtrR may act as a global regulator responsible for preventing intracellular accumulation of toxic chemicals by enhancing the RND efflux system.

**IMPORTANCE:**

Drug efflux protein complexes or efflux pumps are considered as the major determinants of multiple antimicrobial resistance by exporting a wide range of structurally diverse antibiotics in bacterial pathogens. Despite the clinical significance of the increased expression of the efflux pumps, their substrate specificity and regulation mechanisms are poorly understood. Here, we demonstrated that VexAB-TolC, a resistance-nodulation-cell division (RND) efflux pump of *V. cholerae*, is responsible for the resistance to artemisinin (ARS), an antimalarial drug with bactericidal activity. Furthermore, we newly identified AtrR, a TetR family repressor, as a global regulator for VexRAB and the common outer membrane channel, TolC, where VexR functions as the pathway-specific regulator of the vexAB operon. Our findings will help improve our insight into a broad range of substrate specificity of the VexAB-TolC system and highlight the complex regulatory networks of the multiple RND efflux systems during *V. cholerae* pathogenesis.

## INTRODUCTION

The global spread of antimicrobial resistance (AMR) has become a major public health burden leading to failures in the control of infectious diseases caused by bacterial pathogens (1). The emergence of AMR bacteria is the natural process in evolutionary adaptation to antibiotic exposure that threatens bacterial survival and drives selective pressure. The molecular mechanisms of AMR, mostly acquired by inherited mutations to reduce the effectiveness of antibiotics, include (i) modification or protection of antibiotic target sites, (ii) direct inactivation of the antibiotic by hydrolysis or chemical modification, and (iii) prevention of antibiotic access to the target site by reducing membrane permeability or enhancing efflux (2). In particular, the efflux pumps, which actively export broad range of structurally unrelated antibiotics, are considered major contributors to the multidrug resistance (MDR) in bacterial pathogens (3).

In Gram-negative bacteria, tripartite efflux systems of resistance-nodulation-cell division (RND) superfamily are well studied for clinically relevant MDR efflux transporters (2). The RND family pumps consist of three components: the inner membrane protein that functions as the substrate-proton antiporter, the periplasmic membrane fusion protein as the adapter, and the outer membrane efflux protein (OEP) as the channel. The assembly of these components through cell membrane allows expelling the substrates to the extracellular environment by using proton motive force as energy source (4). Based on this mechanical function, RND efflux pumps play a key role in resistance to antibiotics, toxic metabolic intermediates, heavy metals, and solvents. In addition to its role for antibiotic resistance, the multiple functions of RND efflux system have been proposed that are involved in plant-bacteria interaction, bacterial virulence, and biofilm formation (5–7).

We previously reported that an antimalarial drug, artemisinin (ARS), displayed antibacterial activity in combination with copper supplement (8). Interestingly, *V. cholerae* was significantly susceptible to ARS without copper supplementation, whose minimal inhibitory concentration (MIC) is 3.13 µg/mL. ARS is a sesquiterpene lactone compound with endoperoxide bridge of its 1,2,4-trioxane moiety as a pharmacophore (9). The ARS-derived reactive oxygen species (ROS) and/or the carbon-centered organic radicals via endoperoxide bridge cleavage have the potential to accelerate the clearance of the parasite *P. falciparum* from the blood (10, 11). We have demonstrated that copper-mediated ROS generation and subsequent DNA damage might be the key to the antibacterial activity of ARS (8, 12).

In this study, we isolated three ARS-resistant (*atr*) mutants to explore further antibacterial mechanism of ARS in *V. cholerae*. All three *atr* mutants carry mutations in a gene (VCA0767), which encodes a TetR family transcriptional regulator with previously undefined roles in *V. cholerae*. Here, we named this gene as *atrR* (artemisinin resistance regulator). Transcriptomic and mutational analysis revealed that efflux of ARS through upregulation of VexRAB operon was responsible for the ARS resistance of the *atrR* mutant. Based on these results, we presented a new global regulator, AtrR, which directs the expression of VexRAB, where VexR is the pathway-specific regulator of VexAB system. This provides new insights into layered regulatory mechanisms of clinically important RND efflux pump in bacteria.

## RESULTS

### Three *atr* (artemisinin-resistant) mutants were identified

**Fig 1:**
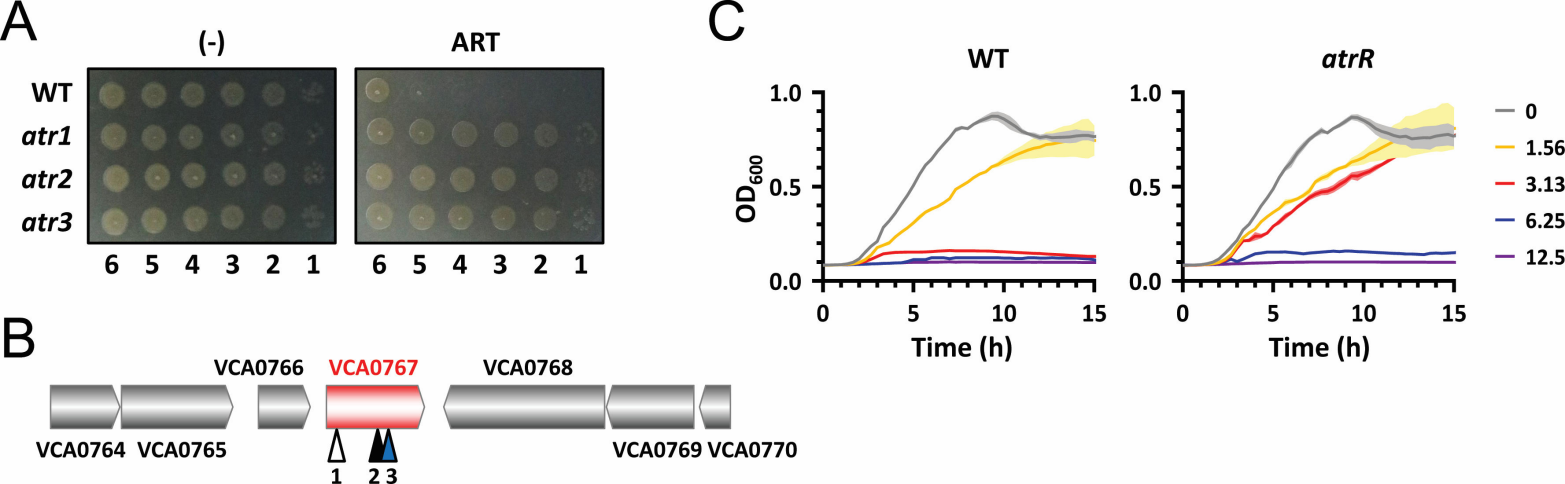
Identification and characterization of *atr* mutants. (**A**) Resistance of the spontaneous *atr* mutants to ARS. Tenfold serial dilutions of the wild-type (WT) and mutant (*atr1-atr3*) cells were spotted onto Luria-Bertani (LB) agar plates with (ART) or without (-) 12.5 µg/mL artesunate. The numbers designate the log(cfu) of the applied cell spots. (**B**) Schematic representation of the mutation sites in the *atr* mutants. The relative positions of the three mutation sites (1–3 for *atr1-atr3*) are indicated under the VCA0767 coding region. The mutations are summarized in Table S1. (**C**) Susceptibility of the *atrR* in-frame deletion mutant to ARS. The culture suspensions (5 × 10^5^ cfu/mL) were incubated in LB broth amended with nothing or artesunate (1.56, 3.13, 6.25, or 12.5 µg/mL), and the growth was semi-continuously monitored by OD_600_ measurement as described in Materials and Methods.

As described above, we previously reported that *V. cholerae* was susceptible to ARS and artesunate (ART) treatment without copper supplementation (8). Based on this finding, we have attempted to investigate the mechanisms of antibacterial activity and/or resistance of ARS in *V. cholerae* as a tractable bacterial system. In the first place, we started to obtain ARS-resistant mutants by spontaneous mutations on the plates containing the lethal amount of ART and then identified three artemisinin-resistant (*atr*) mutants (*atr1-atr3*) with the mutation frequency of 10^−9^ (Fig. 1A). Whole genome sequencing of the three *atr* mutants revealed multiple mutations in each mutant (three for *atr1*, three for *atr2*, and two for *atr3*) (Table S1). Surprisingly, all the three *atr* mutants had missense or frameshift mutations in a common gene, VCA0767, on chromosome II (Fig. 1B): 1-bp deletion at the 15th nucleotide of the gene for *atr1*, 1-bp insertion at the 452nd nucleotide for *atr2*, and CAC to CCC at the 172nd histidine codon (i.e., H172P) for *atr3*. The VCA0767 gene is predicted to encode a TetR family transcriptional regulator, and thus, we renamed this gene *atrR*. It was also observed that the isolated *atr* mutants displayed attenuated virulence in the *Drosophila* systemic infection model (Fig. S1A), although the mechanism by which AtrR directs the virulence pathways needs to be further investigated.

### Loss of VCA0767 (*atrR*) in the *atr* mutants leads to ARS resistance and virulence attenuation as well

To verify the involvement of VCA0767 in ARS resistance and virulence attenuation, we created an in-frame deletion mutant. The MIC value of ARS was determined as 3.13 µg/mL for the wild-type (WT) *V. cholerae*, while that for the *atrR* in-frame deletion mutant was twofold increased as well as for the three *atr* mutants (Table 1). We also compared ARS susceptibility of the wild type and the *atrR* in-frame deletion mutant by determining their growth curve by ARS treatment at various concentrations (0, 1.56, 3.13, 6.25, and 12.5 µg/mL), which corresponded to 0, 0.5, 1, 2, and 4× MIC, respectively. As shown in Fig. 1C, the growth of the *atrR* in-frame deletion mutant was not affected at by 3.13 µg/mL of ARS, unlike that of the wild type. It is noted that the virulence of the *atrR* deletion mutant was also attenuated (Fig. S1B). These results suggest that the ARS resistance and the attenuated virulence of the isolated *atr* mutants might be due to the loss of function of the *atrR* gene.

**TABLE 1:** MICs of ARS

Strain	MIC (μg/mL)
WT	3.13
*atr1*	6.25
*atr2*	6.25
*atr3*	6.25
*atrR*	6.25
*tolC*	0.8
*vexB*	0.8
*vexR*	0.8
*atrRvexB*	0.8
*vexBtolC*	0.8
*atrRvexR*	0.8

### ARS-induced ROS formation and DNA damage are reduced in *atrR* mutant

**Fig 2:**
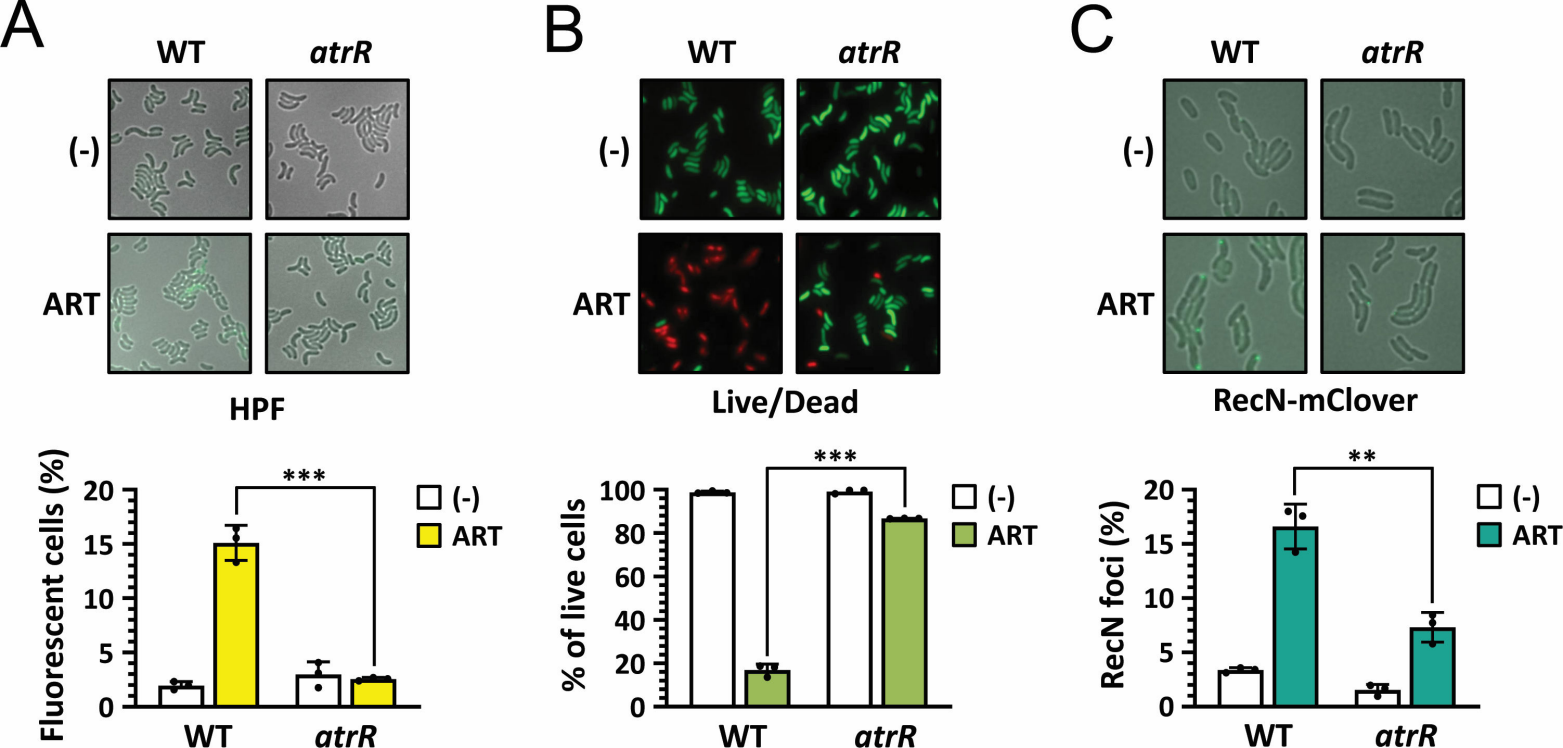
ARS resistance of *atrR* mutant. (**A**) ARS-induced ROS generation. The log-phase cells (5 × 10^5^ cfu/mL) of the WT and the mutant (*atrR*) bacteria were incubated for 3 h in M9 minimal media containing hydroxyphenyl fluorescein (HPF), with nothing (-) or artesunate (313 µg/mL). ROS generation is analyzed by the counts of the cells with the HPF fluorescence, and the fractions of the fluorescent cells in three independent fields are shown in bar graphs, with the error bars representing the standard deviations. Statistical significance between the groups is indicated, based on a *P* value of less than 0.001 (***) by Student’s *t* test. (**B**) ARS-induced bacterial killing. The log-phase cells of the WT and the *atrR* mutant bacteria were pulsed-treated with artesunate (313 µg/mL) and then stained using the mixture of SYTO9 and propidium iodide. Live cells are analyzed by the counts of the cells with the SYTO9 fluorescence, and the fractions of the fluorescent cells in three independent fields are shown in bar graphs, with error bars and statistical significance (***) as in **A**. (**C**) ARS-induced DNA damage. The log-phase cells of the WT and the *atrR* mutant bacteria were treated with artesunate (31.3 µg/mL) for 30 min. The DNA damages were monitored as the foci formation by RecN-mClover3. The foci-positive cells were counted as the cells with DNA damage, and their fractions in three independent fields are shown in bar graphs, with error bars in **A**. Statistical significance is indicated, based on a *P* value of less than 0.005 (**) by Student’s *t* test.

Our previous study suggests that ROS generation and/or DNA damage might be the key to the bactericidal activity of ARS (8). Thus, we next assessed whether the *atrR* mutation leads to reduction in ARS-mediated ROS generation and subsequent DNA damage. The intracellular ROS generation by ARS treatment was examined by staining the bacterial cells with hydroxyphenyl fluorescein (HPF), a hydroxyl radical indicator. As shown in Fig. 2A, HPF fluorescence was increased in the wild type upon ARS treatment, but the fluorescence was significantly reduced in the *atrR* mutant. We also confirmed that the ART-mediated *V. cholerae* killing was significantly reduced in the *atrR* mutant, as visualized by LIVE/DEAD *Bac*Light viability staining (Fig. 2B). These results suggest that the reduced bactericidal activity of ARS in the *atrR* mutant might be due to the reduced ROS generation by ARS treatment.

We further examined whether ARS-induced DNA damage is also impaired in the *atrR* mutant. Based on the formation of damage-inducible repair foci recruiting RecN, a cohesion-like protein, on the double-strand break regions, we visualized the DNA-damaged cells by using the RecN-mClover3 fusion as described in our previous study (8). As shown in Fig. 2C, the RecN foci formation was evidently observed in the wild-type cells upon ARS treatment, whereas that the RecN foci were significantly reduced in the *atrR* mutant. These results suggest that ARS-mediated ROS formation and the subsequent DNA damage could be mitigated by the loss of function of the *atrR* gene.

### VexAB-TolC efflux system and its regulator VexR are upregulated in *atrR* mutant

To gain a mechanistic insight into the ARS resistance directed by the *atrR* deletion, we initially compared the transcriptome profiles of the wild type and the *atrR* mutant. RNA-seq analysis revealed that the known RND efflux pump operon (*vexRAB*) and the outer membrane component (*tolC*) were upregulated in the *artR* mutant (Fig. S2). Although there are six different operons for RND efflux pumps, all the pumps are known to operate with one efflux protein, TolC in *V. cholerae* strains (13, 14). It should be noted that the other five RND efflux systems as well as the four TolC paralogs (VC1409, VC1565, VC1607, and VC1621) were not upregulated at all by ARS treatment (Fig. S2B and C).

**Fig 3:**
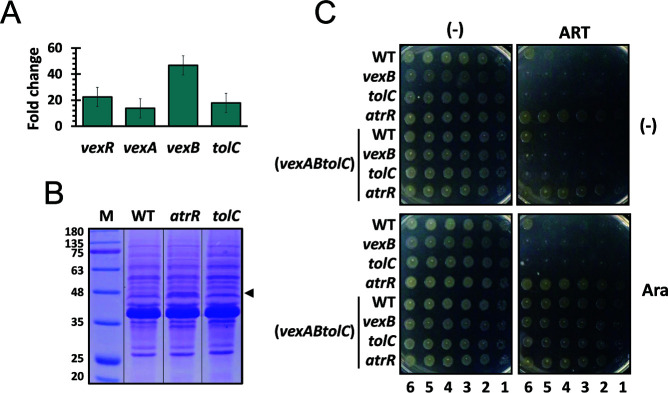
Expression levels of the VexAB-TolC in *atrR* mutant. (**A**) The relative RNA levels of the four genes (*vexR*, *vexA*, *vexB*, and *tolC*) from the logarithmic-phase cells were analyzed by RT-qPCR. The ΔΔC_T_ values of those genes with the *rpoA* mRNA level were compared between the wild type and the *atrR* mutant bacteria. The relative levels in the *atrR* mutant are shown with the standard deviations from three biological replicates. (**B**) Membrane fractions were prepared from the late logarithmic-phase cells of the WT, the *atrR* mutant, and the *tolC* mutant. The protein profiles were analyzed by SDS-PAGE followed by Coomassie brilliant blue R staining. The arrowhead (~47.8 kDa) indicates the TolC protein. (**C**) Resistance of the cells overexpressing the VexAB-TolC efflux pump to ARS. The log-phase cells of the WT and mutant (*vexB*, *tolC*, and *atrR*) bacteria were used, which harbor either pBAD24 alone (control) or pBAD24-derived overexpression of the *vexABtolC* genes. Tenfold serial dilutions of each cell were spotted onto LB agar plates with nothing (-) or artesunate (12.5 µg/mL) (ART) and/or 0.2% L-arabinose (Ara). The numbers designate the log(cfu) of the applied cell spots.

To verify the RNA-seq results, we assessed the transcription levels of the genes (*vexR*, *vexA*, *vexB*, and *tolC*) by quantitative reverse transcription-PCR (RT-qPCR). As expected, *vexRAB* operon and *tolC* gene transcripts were even more (i.e., >10-fold) higher in the *atrR* mutant than in the wild type (Fig. 3A). We also verified the elevated expression of TolC protein from the membrane fraction in the *atrR* mutant bacteria (Fig. 3B). These results suggest that AtrR might act as a negative regulator of the *vexRAB* operon and the *tolC* gene for the sole OEP component for the RND efflux pumps in *V. cholerae*.

### Expression of VexAB-TolC is necessary and sufficient for ARS resistance presumably by exporting ARS

To assess whether the upregulation of VexRAB and TolC is required for the ARS-resistant phenotype of the *atrR* mutant, we created the in-frame deletion of each gene (*vexR, vexB*, or *tolC*). The deletion mutants (*vexR*, *vexB*, and *tolC*) showed hypersusceptible to ARS as assessed by MIC measurement for *vexR* (Table 1) and spotting assay for *vexB* and *tolC* (Fig. 3C). Furthermore, as also shown in Fig. 3C, the inducible overexpression of VexAB and TolC based on the *araBADp* promoter provided ARS resistance to the wild type as well as the *vexB* and the *tolC* mutant bacteria, confirming that the overexpression of the VexAB-TolC efflux system phenocopied the *atrR* mutant. These results suggest that the VexAB-TolC efflux system is necessary and sufficient for the ARS resistance in *V. cholerae*.

**Fig 4:**
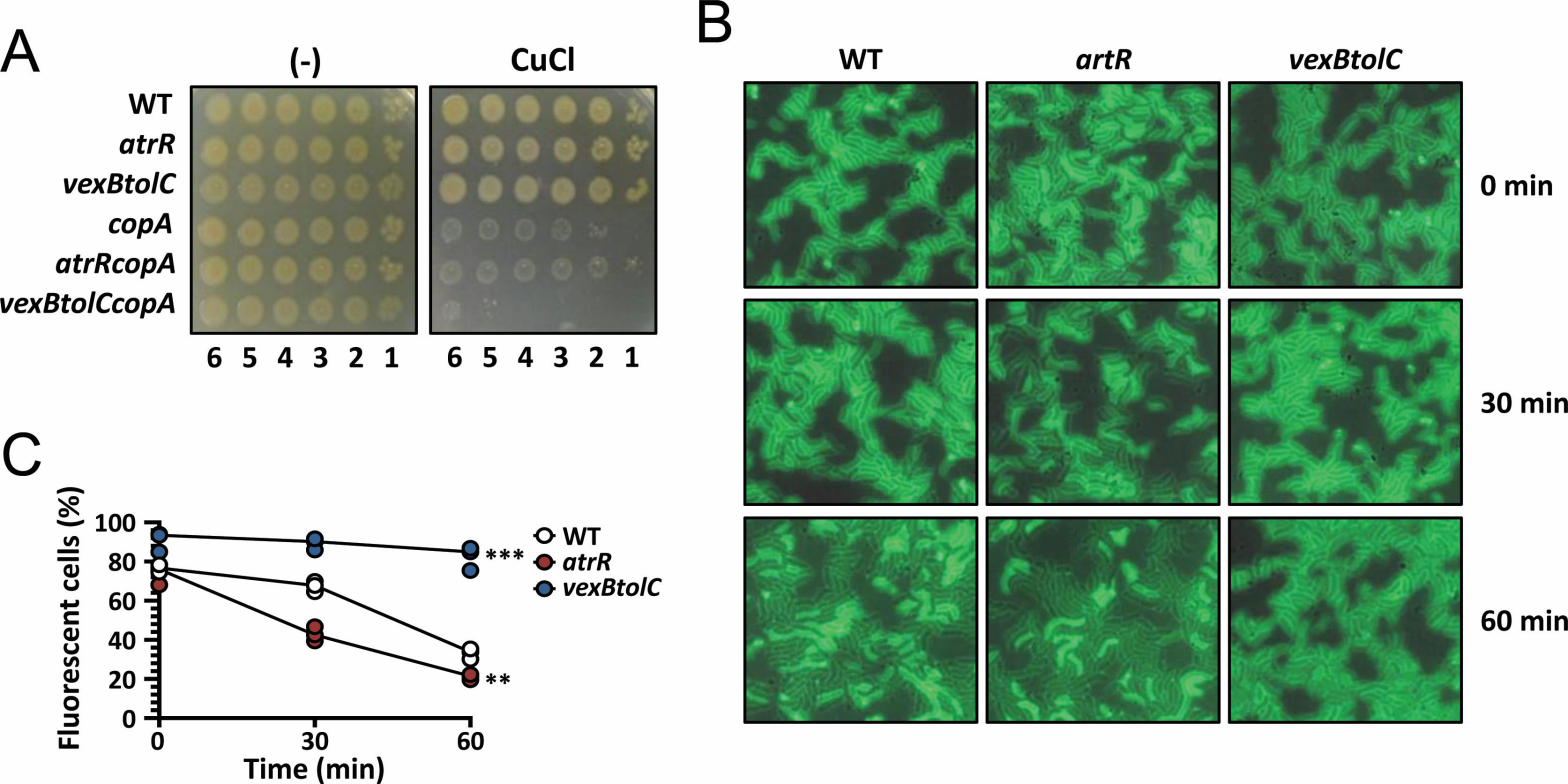
ARS accumulation in *atrR* mutant. (**A**) Susceptibility of the mutants to copper stress. The log-phase cells of WT and the mutants (*atrR* and *vexBtolC*) as well as their isogenic *copA* mutants (*copA*, *atrRcopA*, and *vexBtolCcopA*) were tested. Tenfold serial dilutions of the cells were spotted onto either an LB agar plate (-) or an LB agar plate with 300 µM CuCl. The numbers designate the log(cfu) of the applied cell spots. (**B**) ARS accumulation in the mutants. Each log-phase cell (WT, *atrR*, and *vexBtolC*) was incubated with ARSF, and the ARSF fluorescence was monitored at indicated time (0, 30, and 60 min) under the fluorescence microscope as described in Materials and Methods. (**C**) Quantitation of ARS accumulation in B. The fluorescent cells were counted as the cells with ARS accumulation, and their fractions in three independent fields are shown in line graphs. Statistical significance is indicated, based on a *P* value of less than 0.005 (**) and 0.001 (***) by Dunnett’s multiple-comparison test.

Based on our previous observation that the bactericidal activity of ARS requires the higher level of copper ions (8), we hypothesized that the VexAB-TolC efflux pump might be able to export either copper or ARS. Although *V. cholerae* exploits the copper exporting P-type ATPase (CopA) as the major copper efflux system, we investigated whether the VexAB-TolC system is involved in copper resistance. As shown in Fig. 4A, neither the *vexBtolC* nor the *atrR* mutant displayed altered copper stress phenotypes, whereas the *copA* mutant was susceptible to copper treatment. It should be noted, however, that in the *copA* mutant background, the *atrR* mutant (i.e., *atrRcopA* double mutant) was more resistant to copper, whereas the *vexBtolC* mutant (i.e., *vexBtolCcopA* triple mutant) was even more susceptible. Therefore, it is evident that AtrR might have some physiological role in the copper resistance only in the condition that CopA could be compromised.

This hypothesis prompted us to examine whether the VexAB-TolC efflux system could pump out ARS. We previously synthesized an acridine-linked fluorescent ARS derivative (ARSF), which showed compatible bioactivity to ARS (8). ARSF rapidly penetrates the bacterial cell membrane, with the ARSF-derived green fluorescence detected in the cytoplasm within 5 min and kills *V. cholerae* cells within 30 min in Luria-Bertani (LB) media (presumably in copper-replete condition). Thus, we observed the ARSF fluorescence in M9 minimal media without copper addition, which could be done at 30 and 60 min after ARS treatment. Figure 4B revealed that cytoplasmic accumulation of the ARS fluorescence did markedly disappear in the *atrR* mutant and to the lesser extent in the wild-type cells. It is more remarkable that the fluorescence did not disappear at all in the *vexBtolC* mutant (Fig. 4B and C). Taken together, we suggest that AtrR regulates VexAB as well as TolC, which constitute an RND efflux system required for export of metabolites including ARS to minimize their cytotoxicity.

### VexR is required to resist multiple toxic chemicals

**Fig 5:**
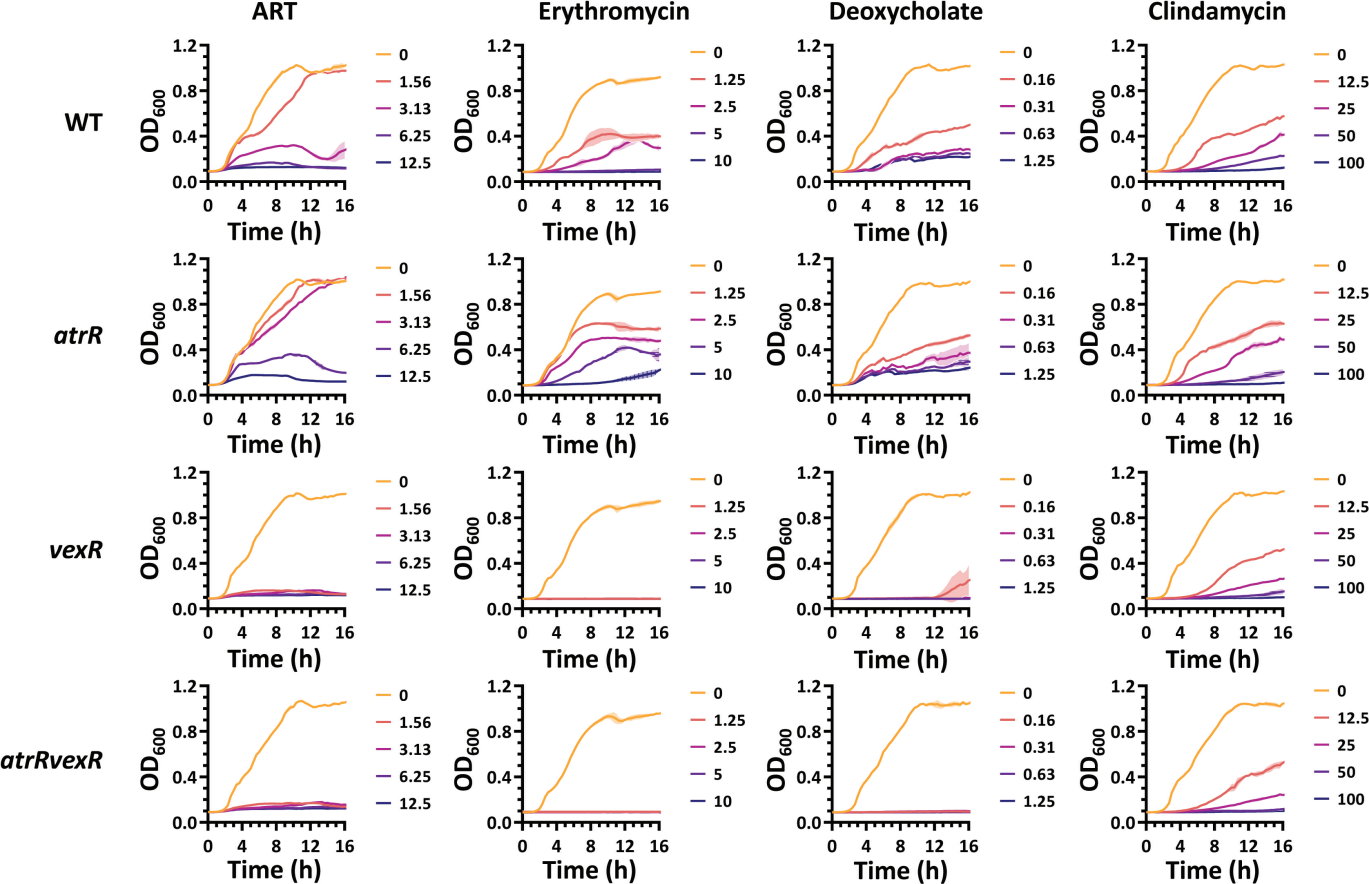
Resistance of *atrR* and *vexR* mutants to toxic chemicals. The log-phase cells of WT and the mutants (*atrR*, *vexR*, and *atrRvexR*) were used to inoculate LB broth containing either nothing or toxic chemicals such as artesunate (ART), erythromycin, deoxycholate, and clindamycin at the indicated concentrations (μg/mL). The growth was semi-continuously monitored by OD_600_ measurement as described in Materials and Methods.

The results and the fact that VexR is the pathway-specific positive regulator of the *vexAB* operon (15) had led us to examine whether the AtrR-mediated *vexAB* regulation requires the functional VexR protein. We created double mutant for both *atrR* and *vexR* (*atrRvexR*). As shown in the MIC determination (Table 1) and the growth curve (Fig. 5), the *atrRvexR* mutant was no less resistant to ARS than the *vexR* mutant, suggesting that *vexR* is epistatic to *atrR* in ARS-resistant phenotype.

**Fig 6:**
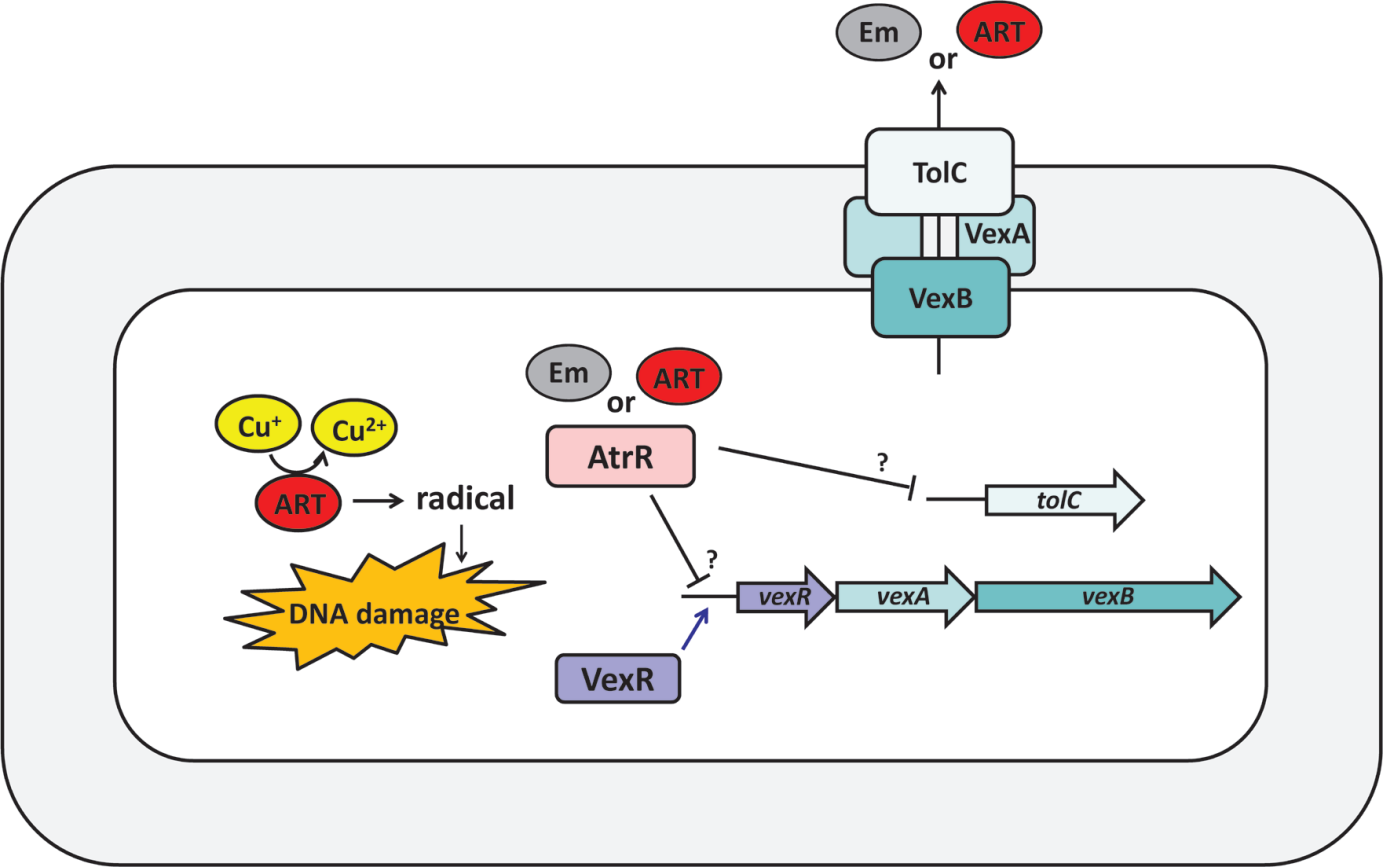
Proposed model for the role of AtrR in resistance to toxic chemicals. Copper-mediated catalytic cleavage of ARS/ART generates carbon-centered ARS radicals, which results in ROS generation in the presence of O_2_. These toxic products may lead to DNA damage. Accumulation of toxic chemicals such as ARS and erythromycin (Em) might be able to trigger inactivation of AtrR, the repressor of the *vexRAB* operon, and the unliked *tolC* gene. VexR is the positive regulator of the *vexRAB* operon for the inner membrane transporter and the periplasmic adaptor, whereas the outer membrane channel encoded by *tolC* is directly regulated by AtrR. The VexAB-TolC efflux pump is involved in the export of the triggering toxic chemicals (ARS and Em).

Figure 5 also revealed that the effect of *atrR* and *vexR* mutation on the role of the VexAB-TolC efflux system. It is well known that one of the roles that the VexAB-TolC efflux system plays is to prevent accumulation of potentially toxic metabolites that are either endogenously generated or exogenously provided (15). We determined the growth curve of the single (*atrR* and *vexR*) and the double (*atrRvexR*) mutants in the presence of the toxic chemicals including erythromycin, deoxycholate, and clindamycin. Despite the differences between the resistance phenotypes upon the chemicals, it is generally acceptable that *vexR* is epistatic to *atrR* in those phenotypes in that those phenotypes of the *vexR* and the *atrRvexR* mutants were indistinguishable in our experimental conditions. It is also evident that AtrR might play a clear role in ARS resistance but no role in clindamycin resistance, whereas VexR might play a significant role in resistance to ARS, erythromycin, and deoxycholate but minute (if any) role in clindamycin resistance. As modeled in Fig. 6, the native role of the VexAB-TolC efflux pump and its regulation by AtrR and/or VexR will be further investigated.

## DISCUSSION

We previously demonstrated that ARS displayed bactericidal activity against *V. cholerae* (8). This apparent antibacterial activity of ARS in *V. cholerae* has prompted us to elucidate the precise mechanism by which *V. cholerae* orchestrates cellular physiology in response to this toxic chemical. In this study, we have isolated spontaneous mutants (*atr1-atr3*), which were resistant to the lethal treatment of ARS and, notably, attenuated in virulence. These mutants have different mutations for a gene (VCA0767 and renamed as *atrR*). This gene encodes a TetR family transcriptional repressor and recently identified as a gene, whose loss of function could lead to resistance to an anti-vibrio hit (16). Although the physiological function of *atrR* still remains elusive, it is noted that the dysfunction of AtrR led to resistance to ARS as well, which is structurally different from the anti-vibrio hit. The resistance to toxic chemicals in the *atrR* mutants could be attributed to the elevated levels of the VexAB-TolC RND efflux system through VexR, in that the overexpression of this efflux system was sufficient to provide the ARS resistance in *V. cholerae*. Despite the opposite effect of AtrR and VexR on the expression of the VexAB-TolC system, the dysfunction of either AtrR or VexR results in attenuated virulence of *V. cholerae*. This suggests that the coordinated or balanced expression of the RND efflux systems or other unidentified targets might contribute to the virulence pathways of this bacterium. The impact of this efflux system is shown to be associated with toxic chemicals rather than copper, given that the *atrR* mutant is also resistant to erythromycin, a bacteriostatic antibiotic and no more resistant to the lethal treatment of copper ions in the presence of functional CopA, the major copper-exporting ATPase.

Taylor et al. (17) initially characterized VexAB as the major efflux system among the six RND efflux systems in *V. cholerae*, which played a predominant role in the export of toxic metabolites and subsequently in intrinsic antimicrobial resistance of *V. cholerae*. The six RND efflux systems [VexAB, VexCD (BreAB), VexEF, VexGH, VexIJK, and VexLM] have been characterized in *V. cholerae* (13, 18). All these efflux systems possess no outer membrane channel or OEP linked in their operon and thus recruit the common unlinked OEP, TolC, for their channel (13). The mutational studies revealed that those RND efflux systems contribute to antimicrobial resistance, intestinal colonization, and expression of virulence factors such as cholera toxin (CT) and the toxin-coregulated pilus (TCP) (19). Importantly, the VexAB-TolC efflux pump is required for resistance to bile salts and multiple antimicrobials such as ampicillin, erythromycin, novobiocin, and polymyxin B *in vitro* (3). Although the involvement of VexAB system in *V. cholerae* pathogenesis has not yet been fully understood, VexAB system was upregulated *in vivo* infection models and was also required expression of virulence factors, CT and TCP (5).

While the regulation of RND efflux system is typically repressed by TetR family transcriptional regulator, the VexAB system is positively regulated by VexR, the pathway-specific regulator co-linked with the *vexAB* genes (15). Therefore, this study reveals a new TetR family transcriptional regulator of this operon by repressing the expression of VexR and TolC in response to ARS and/or presumably other toxic chemicals. Considering that VexR itself exerts positive regulation of the *vexRAB* operon, it needs to be further elucidated, which is the real sensor of ARS and/or other toxic chemicals such as erythromycin between AtrR and VexR. As in the case of the Cpx system that regulates both VexAB and VexGH systems in response to toxic metabolic by-products (15), it should be also noted that AtrR is another regulator that affects the expression of TolC, the only OEP commonly used for the other RND efflux systems, which is also under the control of CpxR (20). TolC is also required for the expression of the ToxR regulon, which is important for virulence potential in *V. cholerae* (21). In this regard, the finding of AtrR as a second global regulator of the RND efflux systems provides an insight into the complex regulatory networks that modulate the expression of the RND efflux systems in response to the broad range of toxic metabolites and chemicals like antibiotics. Future research will be focused on elucidating how AtrR and VexR transduce the signals to regulate the target RND efflux systems, which can be facilitated by computer-assisted *in silico* analyses based on the sequence and the structural similarities in TetR family regulators. A deeper understanding of the regulatory mechanism by which AtrR governs the expression of TolC will lead us to uncover the physiological roles of AtrR as well as the raison d'être of the multiple RND efflux systems in the bacterial pathogenesis.

## MATERIALS AND METHODS

### Bacterial strains and culture conditions

The bacterial strains used in this study are listed in (Table S1) and were grown at 37°C using LB (1% tryptone, 0.5% yeast extract, and 1% NaCl) broth, Müller-Hinton (MH) broth, and M9-glucose minimal medium (1.2% Na_2_HPO_4_, 0.3% KH_2_PO_4_, 0.05% NaCl, 0.1% NH_4_Cl, 2 mM MgSO_4_, 0.1 mM CaCl_2_, and 0.4% glucose) or on 2% Bacto agar-solidified LB plates. Overnight-grown cultures were used as inoculum (1.6 × 10^7^ cfu/mL) into fresh medium and grown at 37°C in a shaking incubator until the logarithmic growth phase (i.e., OD_600_ of 0.7), and then, the cell cultures were used for the experiments described herein.

### Gene deletion and overexpression

The constructs for *atrR*, *vexR*, *vexB*, and *tolC* deletion were created by four-primer (Table S2) SOEing (splicing by overlap extension) PCR, and the resulting PCR products were cloned into pCVD422. These plasmids were introduced into *V. cholerae* by conjugation, and the double-crossover deletion mutants were obtained by sucrose selection from the single-crossover cointegrates. The resulting deletions were verified by PCR.

For inducible overexpression of VexAB-TolC, the *vexAB* and the *tolC* genes were fused and cloned into pBAD24. The plasmid was extracted and purified and then introduced into *V. cholerae* by electroporation.

### Chemical susceptibility assay

The chemical susceptibility of the *V. cholerae* strains was evaluated as in our previous study (8). Briefly, spotting assay and/or growth assay using a microplate reader (BioTek, USA) were generally used for ARS susceptibility by using LB or MH media and artesunate. The copper susceptibility was evaluated by spotting assay on LB plates containing 300 µM CuCl, whereas the susceptibility to other chemicals (erythromycin, clindamycin, and deoxycholic acid) was enumerated by growth assay in LB broth. MIC determination for artesunate was also performed in MH broth by broth microdilution method according to the NCCLS guidelines (22). The medium was subjected to inoculation with the indicated strains (5 × 10^5^ cfu/mL) that had been grown at 37°C to the logarithmic growth phase and then incubated at 37°C on a rotatory shaker. The MIC values were recorded as the lowest concentration of ARS at which no signs of growth were observed based on the OD_600_ value of less than 0.05 after 18-h incubation.

### Virulence assay

*Drosophila* systemic infection was performed using the *Drosophila melanogaster* strain Oregon R, which were grown in corn meal-dextrose medium [0.93% agar, 6.24% dry yeast, 4.08% corn meal, 8.62% dextrose, 0.1% methyl paraben, and 0.45% (vol/vol) propionic acid]. For *Drosophila* infection, 4- to 5-day-old female flies were infected by pricking using a 0.4-mm needle (Ernest F. Fullam, Inc., USA). The needle was dipped into PBS-diluted bacterial suspension containing bacterial cells (5 × 10^7^ cfu/mL) grown to the OD_600_ of 3.0. Prior to infection, the flies were pretreated with a new medium overlaid with 50 µL of artesunate (0.1, 0.5, 1, or 5 mg/mL) for 18 h. Infected flies were transferred to the medium overlaid with artesunate. Survival rates of the infected flies were monitored for up to 36 h post infection. Flies that died within 6 h were excluded in mortality determination. Each assay was repeated at least five times.

### Fluorescence microscopy

LIVE/DEAD *Bac*Light staining (Invitrogen, USA) and detection of fluorescence generated by ROS formation, RecN-mClover3 foci, and ARSF accumulation were carried out using Axio 5 fluorescence microscope (Carl Zeiss, Germany), as previously described (8). The excitation wavelengths are as follows: SYTO9 (480 nm), propidium iodide (490 nm), mClover3 (488 nm), and ARSF (488 nm). M9 minimal media were used for cell culture for fluorescence microscopy, with copper depleted for ARSF fluorescence detection to avoid the toxic activity of ARSF. To immobilize the bacterial cells, the cell of 1 µL was dropped on low-melting agarose (1%), and it was left for 2 min. This sample was observed using fluorescence microscopy. To reduce the toxic effect of ARSF in *V. cholerae* during the microscopic observation, ARSF fluorescence was observed in copper-free M9 minimal medium.

### Measurement of gene expression

Transcriptome profiling was carried out by RNA sequencing as described elsewhere (23), and the RNA levels were determined by RT-qPCR as described previously (24). Briefly, RNA was extracted from the late-logarithmic growth phase (OD_600_ of 0.7) of *V. cholerae* N16961 and its *atrR* deletion mutant, using RNeasy Mini Kit (QIAGEN, Germany). In addition, eluted RNA was treated with DNase I for 1 h at 37°C. For RNA sequencing, Ribo-Zero Magnetic kit (Epicenter, Inc., USA) was used to remove ribosomal RNA from total RNA (5 µg). Library construction and HiSeq2500 (Illumina, Inc., USA)-based RNA sequencing were carried out by a local company (ebiogen, Korea) for two biologically independent replicates. Sequence reads were aligned using Bowtie2 software, and the differentially expressed genes, DEGs were displayed based on normalized counts. Quantile normalization method was used for comparison between samples. For RT-qPCR, cDNA was generated from 1 µg of RNA samples using a Toyobo ReverTra Ace qPCR RT kit and RT-R primer (Table S2) according to the manufacturer’s instruction. qPCR was performed on each cDNA sample with a Toyobo Thunderbird SYBR qPCR mix kit and RT-F and RT-R primers (Table S2) on the StepOnePlus real-time PCR system (Applied Biosystems). The relative expression levels were calculated as described elsewhere (24).

To examine the membrane-associated RND efflux pumps, the total membrane fractions were prepared from the cells that had been grown to the late-logarithmic growth phase (OD_600_ of 0.7), harvested by centrifugation at 7,000 × *g* for 10 min at 4°C, and then washed twice in 10 mM Tris-HCl (pH 7.5). The cells were disrupted by sonication. After centrifugation to remove cell debris, the supernatant was collected and was further centrifuged at 100,000 × *g* for 10 min to separate the membrane proteins from the soluble ones. The pellet, consisting of the total membrane proteins, was resuspended in 10 mM Tris-HCl (pH 7.5) with 2% Triton X-100. TolC expression was analyzed by SDS-PAGE on 15% acrylamide gel and visualized by Coomassie brilliant blue R. The 47.8-kDa band from the *atrR* mutant was cut and treated for protein identification as described previously (25). The gel piece was digested with trypsin, and the resulting peptides were analyzed by mass spectrometry using a Voyager-DETM STR Biospectrometry Workstation (Applied Biosystems).

### Statistics

Statistical analysis was performed using GraphPad Prism version 8.0 (GraphPad Software, USA). Data for each analysis represent a set of three biological repetitions. Statistical significance between the groups is indicated, based on a *P* value of less than 0.01 (*P < 0.01; **P < 0.005, ***P < 0.001) by using the Kaplan-Meier log-rank test, Student’s *t* test, and Dunnett’s multiple-comparison test. Error bars represent standard deviation.

## Data Availability

The high-throughput sequencing data and the RNA-Seq data have been deposited in the Sequence Read Archive (SRA) (https://www.ncbi.nlm.nih.gov/sra) under the BioProject number PRJNA1026370 and in the Gene Expression Omnibus (GEO) (https://www.ncbi.nlm.nih.gov/geo) under the accession number GSE245142, respectively.

## SUPPLEMENTAL MATERIAL

Supplemental figures and tables msystems.00851-23-s0001.pdf Fig. S1 and S2 and Tables S1 and S2.

RNAseq data file msystems.00851-23-s0002.xlsx RNAseq comparison.

ASM does not own the copyrights to Supplemental Material that may be linked to, or accessed through, an article. The authors have granted ASM a non-exclusive, world-wide license to publish the Supplemental Material files. Please contact the corresponding author directly for reuse.
