# Supplementary material for: A TetR family regulator of an RND efflux system that directs artemisinin resistance in Vibrio cholerae
Source: mSystems. 2023 Dec 19;9(1):e00851-23. doi: 10.1128/msystems.00851-23 (PMC10805010; doi:10.1128/msystems.00851-23)
Supplement: Supplemental figures and tables — Fig. S1 and S2 and Tables S1 and S2. [file msystems.00851-23-s0001.pdf]

## SUPPLEMENTAL DATA

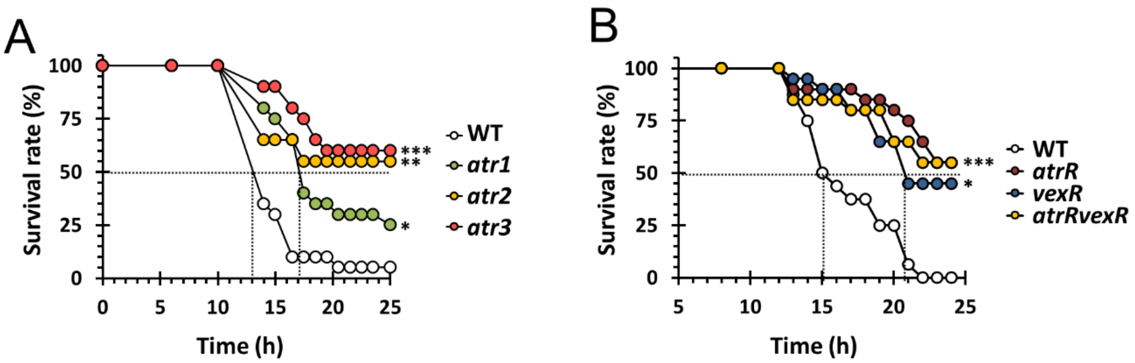


**Fig. S1. Virulence of *atr* mutants in *Drosophila melanogaster*.**

A group of 50 female flies were infected with the stationary-phase cells of the wild type (WT), the spontaneous mutants (*atr1*-*atr3*. **A**), and the in-frame deletion mutants (*atrR*, *vexR*, and *atrRvexR*. **B**) bacteria. Mortality of the infected flies was measured as described elsewhere (2). The dotted lines represent the time required to reach 50% mortality. The statistical significance based on a log-rank test is indicated as follows: *, $p < 0.01$; **, $p < 0.005$; ***, $p < 0.001$.

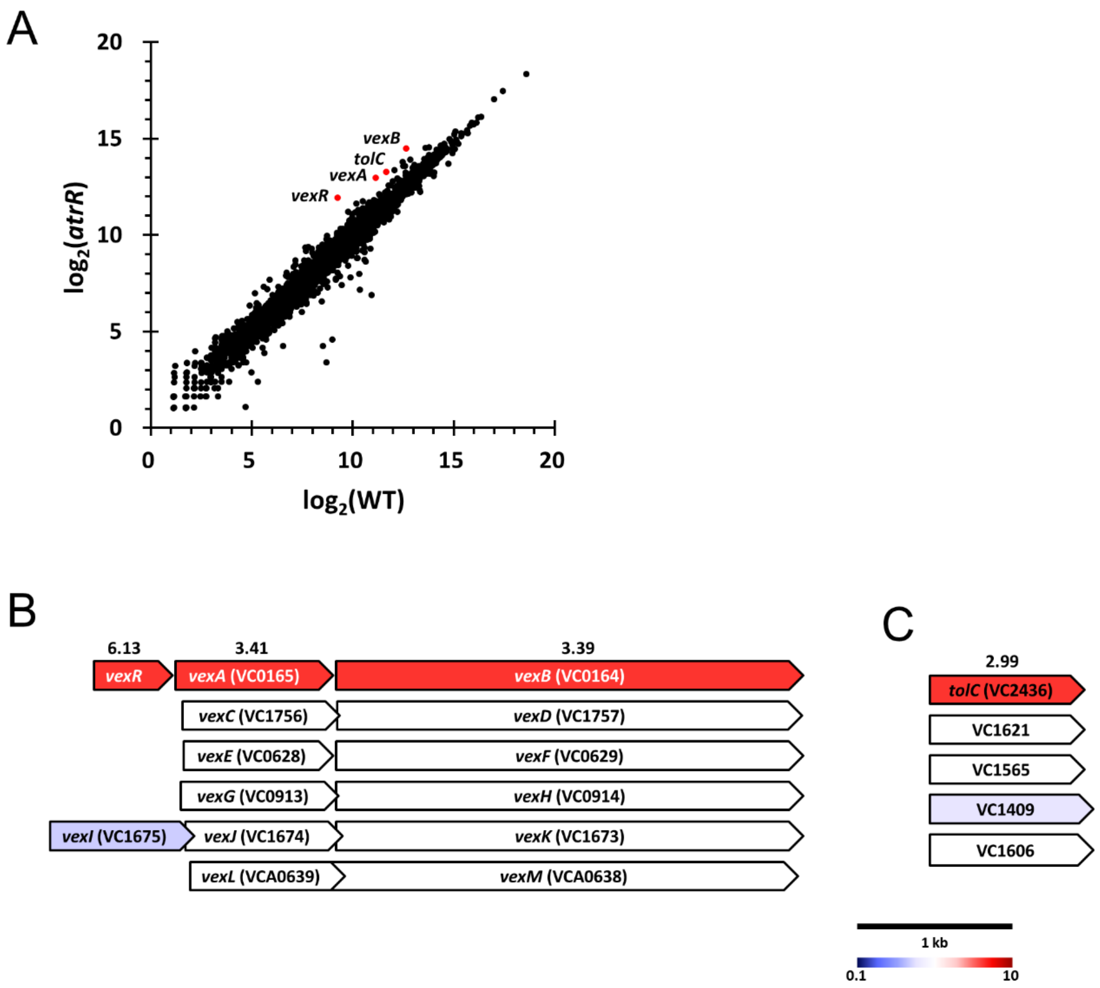


**Fig. S2. Transcriptome of *atrR* mutant.**

A. Scatter plot for the transcriptome of the *atrR* mutant in comparison with that of the wild type (WT). The x-axis represents the normalized data ($\log_2$) from the WT bacteria and the y-axis represents those from the *atrR* mutant bacteria. Red dots indicate the 4 representative genes (*vexR*, *vexA*, *vexB*, and *tolC*) upregulated in the *atrR* mutant, whose induction folds are designated in **B** and **C**.

B and C. Relative expression levels of the 6 RND efflux systems (**B**), the *tolC* gene, and the 4 *tolC*-paralogous genes (**C**). The levels are shown from 0.1 fold (i.e. downregulated;) to 10 fold (i.e. upregulated) with the intensity scale. The four upregulated genes (*vexR*, *vexA*, *vexB*, and *tolC*) in **A** are shown in red with the number indicates the relative levels over those in WT.

## Table S1. Bacterial strains and plasmids used in this study.

| *Strain or plasmid* | *Relevant characteristics or purpose*[a] | *Reference or source* |
|---|---|---|
| <u>*Vibrio cholerae*</u> | | |
| WT | *V. cholerae* N16961 | Lab collection |
| *atr1* | Spontaneous artemisinin resistant mutant of WT; at a site of VCA0767(frameshift) and VC2614(L62F, G142D) | This study |
| *atr2* | Spontaneous artemisinin resistant mutant of WT; at a site of VCA0767, VC2135, and VC2198 (all frameshifts) | This study |
| *atr3* | Spontaneous artemisinin resistant mutant of WT; at a site of VCA0767(H172P) and VC0122(G173S) | This study |
| *atrR* | N16961 with in-frame deletion for *atrR* (VCA0767) | This study |
| *vexR* | N16961 with in-frame deletion for *vexR* | This study |
| *vexB* | N16961 with in-frame deletion for *vexB* | This study |
| *tolC* | N16961 with in-frame deletion for *tolC* | This study |
| *copA* | N16961 with in-frame deletion for *copA* | This study |
| *vexBtolC* | N16961 with in-frame deletions for *vexB* and *tolC* | This study |
| *atrRvexR* | N16961 with in-frame deletions for *atrR* and *vexR* | This study |
| *atrRcopA* | N16961 with in-frame deletions for *atrR* and *copA* | This study |
| *vexBtolCcopA* | N16961 with in-frame deletions for *vexB*, *tolC*, and *copA* | This study |
| <u>*Escherichia coli*</u> | | |
| DH5α | multi-purpose cloning | Lab collection |
| <u>Plasmids</u> | | |
| pBAD24 | Arabinose-inducible overexpression based on *araBADp* promoter; $Cb^R$ | Guzman et al. 1995 [1] |
| pBAD24-*vexABtolC* | pBAD24 derivative for overexpression of *vexABtolC*; $Cb^R$ | This study |
| pBAD24-RecN-mClover3 | pBAD24 derivative for overexpression of RecN fused with mClover3; $Cb^R$ | Chung et al. 2022 [2] |

$Cb^R$, carbenicillin- and ampicillin-resistant;

**Table S2. Primers used in this study.**

| Primer | Oligonucleotide sequence (5´ to 3´) |
|---|---|
| Gene deletion and expression | |
| atrR-UP-F | CCATCTTGCATGCATTCCGCC |
| atrR-UP-R | GATCCGTGCTTTGCCATCCAAAAGGATATGTGCCAT |
| atrR-DN-F | ATGGCACATATCCTTTTGGATGGCAAAGCACGGATC |
| atrR-DN-R | TGTCTAGACTCCGCTGTGCCG |
| vexR-UP-F | GGGAATTCGCCGCTATAATGAAC |
| vexR-UP-R | GTACGGCACCACGTACGTCAGCGCACGCTCAATACG |
| vexR-DN-F | CGTATTGAGCGTGCGCTGACGTACGTGGTGCCGTAC |
| vexR-DN-R | CCCTGCAGCAGCAGGATCGCCATAAG |
| vexB-UP-F | GGTCTAGACATTGTGCGC |
| vexB-UP-R | CAAGGTACCAATTGCCAGGGTAATAGCCGGTGCTCA |
| vexB-DN-F | GTGAGCACCGGCTATTACCTGGCAATTGGTACCTTG |
| vexB-DN-R | CAGCATGCGTAACCATCCGG |
| tolC-UP-F | CTGAATTCGGTACTTCACCATGC |
| tolC-UP-R | TCCATCACGTCTTGCTCACTTAGTGTGCCTAGCGCAGCAC |
| tolC-DN-F | GTGCTGCGCTAGGCACACTAAGTGAGCAAGACGTGATCGA |
| tolC-DN-R | TGTCTAGACGGCAGAGCTTAGTG |
| Gene expression | |
| rpoA-RT-F | TGGTTGCAGGTGACATCACC |
| rpoA-RT-R | ATACGCATAGCGATCGCAGC |
| vexR-RT-F | ATTGAAGTGCGTTTGCACG |
| vexR-RT-R | AGCCACATAGTACGGAAGCG |
| vexA-RT-F | ACGCTACAAGGGTCTGTTCG |
| vexA-RT-R | TCAATATCAGCGGAGAGCGC |
| vexB-RT-F | ATTGCCATCCCAGTTATTGC |
| vexB-RT-R | AACAGTGAGCCGGTGATACC |
| tolC-RT-F | GCGCTAGGCACACTAAGCTC |
| tolC-RT-R | ACTACGGCTAGAAGTCACCG |

## SUPPLEMENTAL REFERENCES

1. Guzman LM, Belin D, Carson MJ, Beckwith J. 1995. Tight regulation, modulation, and high-level expression by vectors containing the arabinose *pBAD* promoter. J Bacteriol 177: 4121-4130. https://doi.org/10.1128/jb.177.14.4121-4130.1995.
2. Chung IY, Jang HJ, Yoo YJ, Hur J, Oh HY, Kim SH, Cho YH. 2022. Artemisinin displays bactericidal activity via copper-mediated DNA damage. Virulence 13:149-159. https://doi.org/10.1080/21505594.2021.2021643.
